# Initiative action of tumor-associated macrophage during tumor metastasis

**DOI:** 10.1016/j.biopen.2016.11.002

**Published:** 2017-01-05

**Authors:** Saroj Singh, Neesha Mehta, Jiang Lilan, Meen Bahadur Budhthoki, Fu Chao, Li Yong

**Affiliations:** Department of Oral and Maxillofacial Surgery, The Affiliated Hospital of Stomatology, Chongqing Medical University, No. 426 Songshibei Road, Yubei District, Chongqing 401147, China

**Keywords:** Tumor-associated macrophage, Th17 & Tregs cells, Inflammation, Metastasis

## Abstract

Tumor-associated macrophages (TAMs) are a significant component of the microenvironment of any solid tumors in the majority of cancers, associated with unfavorable prognosis. TAMs emerge as attractive targets for therapeutic strategies aimed at reprogramming their protumor phenotype into an effective antitumor activity.

In this review article, we present an overview of mechanisms responsible for TAMs recruitment and highlight the roles of TAMs in the regulation of tumor angiogenesis, invasion, metastasis, immunosuppression, and chemotherapeutic resistance. We describe the interplay between Th17 cells and other immune cells in the tumor microenvironment, and we assess both the potential antitumorigenic and pro-tumorigenic activities of Th17 cells and their associated cytokines. Understanding the nature of Th17 cell responses in the tumor microenvironment will be important for the design of more efficacious cancer immunotherapies. Finally, we discuss TAM-targeting therapy as a promising novel strategy for an indirect cancer therapy.

## Highlights

• Macrophage may promote tumor growth and suppress local immunity.
• FoxP3^+^T cells, Th17 cells play dual roles in regulating antitumor immune responses.
• Treat cancer patients by reeducating TAMs to be tumor inhibitory cells.
• High TAM infiltration associated with poor prognosis and therapeutic failure.
• TAMs become the foci of promising novel cancer therapies.

## 1 Introduction

Cancer is a disease characterized by rapid growth of cells in the body, often in the form of a tumor. There was an estimation of 8.2 million deaths from cancer in the world in 2012, of which, 4.7 million (57%) deaths occurred in males and 3.5 million (43%) in females, giving a male: female ratio of 10:8 [1]. According to The National Central Cancer Registry (NCCR) of China, it was estimated that there were 34,319 new cases diagnosed as oral cavity cancer in China. In 2010, among the diagnosed cases, there were 14,652 cases resulting in deaths due to oral cavity cancer. In 2010, the crude incidence and mortality rate of oral cavity cancer was 1.11/100,000, accounting for 0.75% of overall cancer deaths, ranked the 20th in all cancer sites [2].

The innate immune cells that play a broad role in host defense and the maintenance of tissue homeostasis are macrophages [3]. In general, we can classify macrophages into two subsets: the classical M1 and the Alternative M2 macrophages [4]. The phenotype M1 is driven by the Th1 cytokine interferon-*γ*, bacterial moieties such as lipopolysaccharide (LPS), and Toll-like receptor (TLR) agonists. The production of pro-inflammatory factors such as IL-6, IL-12, IL-23, and tumor necrosis factor-α(TNF-α) are their characteristic feature. Conversely, the M2 macrophages exert anti-inflammatory and pro-tumorigenic activities. Within the tumor, macrophages are a major stromal component, where they commonly termed tumor-associated macrophages [5], [6]. The localization of TAMs in a human sample is usually determined by marking the expression of CD163 and CD68 proteins. The infiltration of macrophages is largely related to poor prognosis of malignant tumors [6]. Cancer metastasis or metastatic tumor is a process in which cancer cells spread from the primary tumor (where it started) to different area(s) of the body. Cancer metastasis is the primary cause of morbidity and mortality which is responsible for about 90% of total cancer deaths [7].

This review is aimed to provide an overview of the action, function and reaction of the tumor-associated macrophages during tumor growth, and its role in metastasis process. This review will also throw some lights on anti-cancer therapy and drugs delivery process by TAMs. It is not the intent of this review to provide an in-depth description of each macrophage, types and classification of tumors and metastasis of various tumor as each topic itself can be a lengthy review. It hoped that this review can serve as a lead for readers who are interested in the recent development of the functions of TAMs during tumor metastasis.

## 2 Tumor-associated myeloid cells: differentiation pathways

The cellular content of solid tumors is characterized by the presence of a leukocyte infiltrate including lymphocytes and myeloid cells from early stages. Recent evidence indicates that among leukocytes, myeloid cell populations represent a prominent component, both regarding number and functions, supporting tumor growth and progression and has prognostic value [8]. Tumor-associated myeloid cells (TAMC) contain five distinct myeloid populations- (1) Tumor-associated macrophages (TAM), (2) Monocytes expressing the angiopoietin-2 (Ang-2) receptor Tie2 known as Tie2-expressing monocytes or (TEM), (3) Myeloid-derived suppressor cells (MDSC), (4) Tumor-associated neutrophils (TAN), and (5) Tumor-associated dendritic cells (TADC) [8].

Tumor-associated macrophages are usually the most abundant immune population in the tumor microenvironment due to the early infiltrating leukocyte populations within the tumors [9]. They develop from blood monocytes which actively engaged from the circulation into tumor tissues. Aptly stimulated macrophages can kill tumor cells *in vitro,* shown by early studies; however, TAM, conditioned by the tumor microenvironment, loose the cytotoxic capability and rather exert several pro-tumoral functions, mediating cancer-related inflammation, angiogenesis, immunosuppression, tissue remodeling, and metastasis [9], [10].

TAM is a hallmark of myeloid which shows heterogeneous behavior, and the cells overgeneralized in a polarization concept with two extreme M1 and M2 phenotypes with distinct and somehow contrasting functions [11]. Classically activated or M1 macrophages are bacterial products and Th1 cytokines (e.g., LPS/interferon-γ). M1 macrophages strongly produce inflammatory and immune stimulating cytokines, trigger adaptive responses, secrete reactive oxygen species (ROS) and nitrogen intermediates, and have a cytotoxic effect towards transformed cells. In contrast, alternatively activated or M2 macrophages differentiate in response to Th2 cytokines (e.g., interleukin (IL)-4, IL-13) [12]. In conflict with their M1 counterpart, M2 macrophages produce growth factors, leading to tissue repair and angiogenesis activation, which have high scavenging activity, and also acts as inhibitive adaptive immune responses [5], [8]. As a result, macrophages are a very heterogeneous cell population, which can display different functions depending on the context. Macrophages can be either immunosuppressive which inhibits inflammation or immune stimulatory at the beginning of the inflammatory response [5], [8], [13].

Myeloid-derived suppressor cells (MDSCs) are a heterogeneous population of early myeloid progenitors, immature granulocytes, macrophages, and dendritic cells at different stages of differentiation. It also has the ability to suppress T-cell functions [14]. MDSC accumulate in the blood, bone marrow, and secondary lymphoid organs of tumor-bearing mice. Their presence in the tumor microenvironment has been suggested to have a causative role in promoting tumor-associated immune suppression and local tumor-associated factors promote their activation [15].

MDSC, isolated from blood of patients with glioblastoma, colon cancer, breast cancer, lung cancer, or kidney cancer of human are poorly defined [16], [17]. Recent studies of human MDSC projected that they have a characteristic CD34^+^, CD33^+^, CD11b^+^, and HLA-DR^−^ profile [18]. Likewise, human MDSC is divided into two main subsets: (1) Monocytic MDSC (M-MDSC) which characterized by the expression of CD14, and (2) Granulocytic MDSC (G-MDSC), which is recognized by positivity for CD15.

**Fig. 1:**
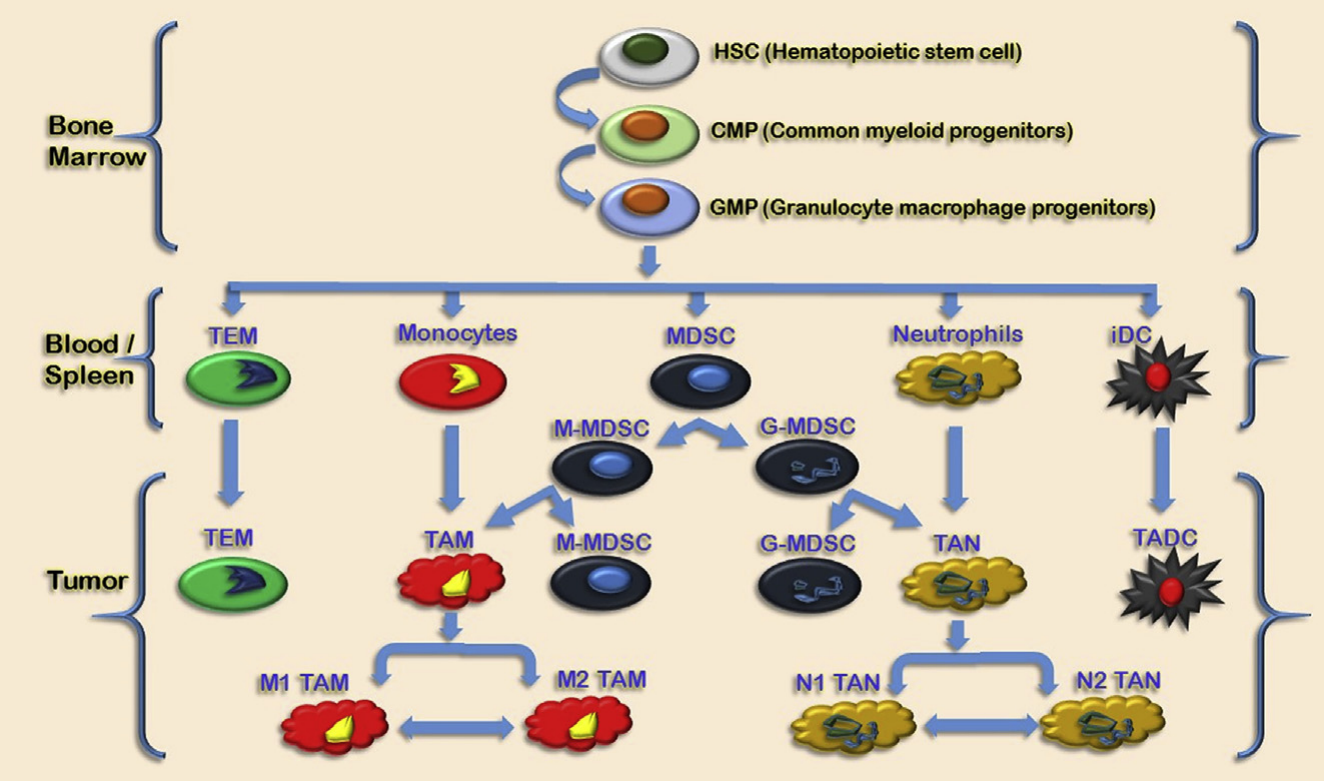
**Tumor-associated myeloid cells: differentiation pathways**. Above figure illustrate that myeloid cells originate from hematopoietic stem cells (HSC) in the bone marrow. HSC in the bone marrow differentiated into various myeloid cell lineages in diverse compartments like bone marrow, blood/spleen, and tumor. In bone marrow, HSC distinguishes into CMP to granulocyte macrophage progenitors (GMC) and in a tumor cell, various macrophages and neutrophils changes to various differently polarized phenotypes like M1-M2 for Tumor-associated Macrophage (TAM) and N1-N2 for neutrophils. (*TEM* – Tie2-expressing monocytes, *MDSC* – myeloid-derived suppressor cells, *M*-*MDSC* myeloid MDSC, *G*-*MDSC* granulocytic MDSC, *TAN* – tumor-associated neutrophils, *iDC* – immature dendritic cells, *TADC* – tumor-associated dendritic cells [8]).

A recent study shows that a small number of dendritic (DC) found in most human and murine neoplasms have an immature phenotype (iDC). Likewise, to macrophages and neutrophils, plasticity is the main feature of these cells. DC localized in different forms in tumors; such as, in breast cancer immature langerin^+^ DC interposed within the tumor mass, whereas more mature CD83^+^, DC-LAMP^+^ DC are limited to the peritumoral area [8], [19] (See Fig. 1).

## 3 Major role of innate immune cells during cancer and anticancer immunity

“The first line of defense” against pathogens and cancers are the innate immune system. They engrossed into the tumor site in any tumor, where they can recognize the transformed cells. Due to the interaction between tumor cells and innate immune cells in the tumor microenvironment, innate immune cells lead to the promotion of tumor growth, angiogenesis, and metastasis. Therefore, before developing any strategies for immunotherapy of cancer, profound knowledge of the innate immune system in tumor immunity and tumorigenesis is a must.

### 3.1 Natural killer (NK) cells

Natural killer (NK) cells are effectors cells which are considered to play a critical role in the early innate immune response to antitumor immunity [20]. Natural killer cells, by their morphology, their expression of lymphocyte markers, and their origin from the common lymphoid progenitor cell in the bone marrow, were qualified as lymphocytes. CXCL12 and CXCL3L1 Chemokines are key factors for NK migration to tumor sites, and play a significant role in the tumor immunosurveillance [21].

NK cells produce several cytokines, such as interferon-γ (IFN-γ), TNF-α, interleukin (IL)-10, and growth factors under some physiological and pathological condition. Furthermore, NK cells also produce many chemokines that impact dendritic cells (DCs), macrophages and neutrophils during an immune response, hence endowing NK cells with regulatory functions. Recent studies revealed that NK cells also have “memory” properties that were previously endorsed only to T and B cells [22]. The interaction between self-MHC molecules and NK cells for making functionally competent are the main focusing point of current challenges. The inhibitory receptor “Ly49C” which is a subset of NK cells interact with self- MHC molecules on target cells and plays a vital role in enabling immature NK cells to develop into functioning mature cells. Thus, Ly49C- positive NK cells are considered “licensed”. Whereas, an immature stage of NK cells with Ly49C-negative is considered “non-licensed” [23]. With the help of these finding we might get answer to the important question – why donor NK cells, which is administrated to leukemia patients, do not show antitumor effects during bone marrow transplantation [24].

The NK cells and dendritic cells (DCs) interaction is essential for the intensification of innate responses and the initiation of potent adaptive immunity. Mature DCs are activated by NK cells through cytokines (TNF-α and IFN-γ) and receptor (NKp30 and NKG2D)-mediated mechanisms, however, Immature DCs are susceptible to NK cell-mediated cytolysis. Effector activities of NK cells, for example, IFN-γ production, proliferation, and cytotoxic activities are triggered by activated DCs. Besides this, treatment with TLR3 agonist polyinosinic-polycytidylic acid (Poly (I: C)) activates DCs to stimulate antitumor activities of NK cells. Therefore, mutual interaction between NK and DC leads to the proper direction and quality of antitumor immunity, which may be the milestone for the development of effective cancer immunotherapy [8], [25], [26].

### 3.2 Natural killer T (NKT) cells

NKT cells are a subset of T cells which share phenotypic and functional characteristics of both NK cells and T cells and express specific NK cell markers such as CD161 or NKR-P1. Even though NKT cells constitute only a small percentage of lymphocytes, they can regulate many other cell types such as macrophages, dendritic cells (DCs), CD8 + T, and NK cells and can play vital roles in many aspects of the immune [27], [28]. NKT cell can be further be divided into, Type I and type II NKT cells. Recent studies show that, Type I and type II NKT cells have not only opposite roles but also counter-regulate each other. This helps to identify that interaction between type I and type II NKT cells may be equivalent to that of Th1 and Th2 cytokines and thus, play different roles in the pathogenesis of many diseases [8], [28].

### 3.3 γδ-T cells

Based on their expression of T cell antigen receptors (TCRs) T cells can be divided into two subsets αβ-T cells and γδ-T cells. Indeed, the αβ-T and γδ-T cell populations distinguish different types of antigens. Non-self-peptide fragments have recognized by αβ T cells which restrict MHC molecules. Conversely, γδ-T cells recognize unconventional antigens which include stress molecules like non-peptidic metabolites of isoprenoid biosynthesis, MICA, and MICB [29], [30].

γδ-T cells shows a variety of functions, such as (1) Production of a wide range of cytokines and chemokines so that it can regulate other immune (e.g. Tregs) and non-immune cells, (2) Provides help for B cells, (3) Trigger dendritic cell (DC) maturation, (4) Involved in the macrophage recruitment, (5) Exhibit varying degrees of cytolytic activity to various kinds of malignancies, such as neuroblastomas, (6) Produce growth factors that maintain epidermal integrity, (7) present antigens for αβ T-cells [29]. Stimulating the adaptive immune system which depends on major histocompatibility complex (MHC)-restricted αβ-T cells are major immunotherapeutic approaches for an anti-tumor response. γδ T cells are considered to be a member of innate immune cells which shows an important role in immune defense and Immunosurveillance against tumors, such as leukemia, neuroblastomas, lymphomas, melanomas, and other types of carcinomas [29], [31].

### 3.4 Macrophages

The macrophage stands a diverse cell type playing a multifaceted role in the innate and adaptive immune response which serves as the first line of defense against tumorigenesis acts by directly killing tumor cells and generating various antitumor mediators [32]. Conversely, macrophages have the ability to acquire invasive and metastatic activities [10].

Generally, “patrolling” and “inflammatory” are two types of monocytes that differ both regarding surface marker expression and function. LY6C^low^ CX3CR1^high^ are an example of patrolling monocytes which protect and repair endothelial surfaces, partially by coordinating neutrophil recruitment. Both LY6C^low^ CX3CR1^high^ are inflammatory monocytes released from spleen and bone marrow into the blood and recruited to tissues by inflammatory stimuli in a CCL2-dependent manner and contribute to anti-infectious control when tissue macrophages alone cannot fight off invading pathogens [33], [34].

Macrophages are released from the bone marrow and differentiated from immature myeloid precursors or circulating monocytes [34]. Immature monocytes are further differentiated into M1 or M2 macrophages. The function of M1 macrophages is to induce an antitumor response by triggering cytotoxic activities and producing IFN-γ and IL-12 [8], [35]. Whereas, the function of M2-polarized macrophages is involved in tissue repair and wound healing, and markers such as IL-10, TGFβ, IL-4 and IL-13 and being involved in tissue homeostasis [36]. Recent study results have emphasized on the integration of M2-polarized macrophages with immunostimulatory pathways to induce differentiation of Treg cells [37], and on the other hand, alternative activation of human mononuclear phagocytes has been induced by Tregs [38]. Therefore, cancer has served as a paradigm of *in vivo* M2 polarization [39].

### 3.5 Dendritic cells

DCs are the professional antigen-presenting cells(APCs) which play a critical role in the regulation of the adaptive immune response in the body against pathogens as well as tumors [40], [41]. Dendritic cells turn as a Toll-like receptor (TLRs) essential for the activation of various effectors [41]. A recent study illustrates, DCs most likely derived from monocytes, induces Th17 cell discrepancy through releases of Th17 cell-polarizing cytokines [42]. Through several balancing mechanisms, DCs capture antigen in peripheral tissue. Antigen-loaded DCs migrate into the draining lymph nodes through the afferent lymphatics after antigen loading. These immature DCs triggers their migration towards secondary lymphoid organs. For the meantime, the proteins convert into peptides that bind to both major histocompatibility (MHC) class I and MHC class II molecules or non-classical MHC molecules of the CD1 family which permit the assortment of rare antigen-specific T-lymphocytes. Now, activated T cells initiate dendritic cells towards terminal maturation of DCs. Efferent T cells further induce by expansion and differentiation of T lymphocytes. DCs remain immature if it does not receive maturation signals, thus, leading to immune regulation and/or suppression [41]. Antigen-specific effector T cells with different functions had differentiated by naive CD4+ T cells and CD8+ T and CD4+ T cells can turn into T helper 1 (TH1) cells, TH2 cells, TH17 cells or T follicular helper (TFH) cells. These cells support B cells to differentiate into antibody-secreting cells, along with regulatory T (Treg) cells that downregulate the functions of other lymphocytes. Effector cytotoxic T lymphocytes (CTLs) has given rise to naïve CD8+ cells [41].

Dendritic Cells also referred as ‘nature's adjuvants' due to its properties. It's used as the natural targets for antigen delivery and acts as a bridge between the innate and the adaptive immune responses that control both tolerance and immune responses [41]. Dendritic cell infiltrated in tumor can induce tumor growth and metastasis by regulating angiogenesis, host immunity, and tumor metastasis. Likewise, DCs cause poor tumor immunogenicity if produced from indoleamine 2, 3-dioxygenase (IDO) and generate Foxp3-positive regulatory T cells after interface with other innate lymphocytes, for example, γδ-T cells and NKT cells [8], [41], [43]. Owing to these significant properties of DCs, used as candidates for antigen delivery and vaccination most essentially used for therapeutic vaccination against cancer [41].

### 3.6 Granulocytes

Granulocytes is a type of immune cell with the key mediators of inflammation having a potential role in the initiation of immune response and cascades against tumors [44]. The release of cathepsin G, azurocidin, reactive oxygen species, and inflammatory cytokines, induced by granulocytes which play a vital role in tumor destruction. In addition, granulocytes, along with macrophages and T cells, are key effectors that elicit antitumor responses using DNA vaccines in murine tumor models [45]. Furthermore, Clinical responses of GM-CSF-secreting cancer cells and Bacillus Calmette-Guerin (BCG) is a result of dense infiltration of granulocytes in tumor tissues which is found in patients with advanced melanoma and bladder carcinoma, respectively [46]. Alternatively, secretion of proteinases, ROS, and cytokines are the result of granulocytes contribution towards tumor angiogenesis and metastasis that may act as antitumor effectors in different conditions [47]. Thus, depending on the distinct environment, granulocytes show both pro- and antitumor activities.

### 3.7 Th17 and Tregs cells in tumor: generation and migration

In inflammation, favorable host response to infection will take no time to contribute to inflammatory disease, if unregulated. Severe human inflammatory diseases can caused by the Th17 lineage of T helper (T) cells. These cells exhibit both instability and plasticity upon re-stimulation [48]. In Addition, even though TH17 cell instability or plasticity had associated with pathogenicity, it is unidentified whether this feature could present a therapeutic opportunity, whereby previously pathogenicTH17 cells could adopt an anti-inflammatory outcome [48].

Tumors which formed in immunologic tolerance tissue environments, are naturally less immunogenic than normal and uses numerous mechanisms to suppress the generation of effector T cells [49]. Tumors usually maintain production of immunologic tolerance environments to avoid antitumor immune responses and harbor high numbers of Forkhead box P3(FoxP3^+^) T cells (commonly called Tregs). FoxP3^+^ Tregs made in the human immune system (Thymus) as natural FoxP3^+^ T cells. Origin of Treg cells and their persistence induction in the periphery from naïve CD4^+^ T cells. Moreover, IL-10-producing Tregs (Tr1 cells) are made from naïve CD4^+^ T cells. Tregs produce suppressive cytokines, for example, IL-10, IL-35, and TGF-β [50]. For the prevention of autoimmune diseases, Tregs play vital roles. Every time effector T cells formed during immune responses, Tregs are made.

Effector T cells and Tregs initiation occurs mainly in secondary lymphoid tissues. This is because naive CD4^+^ T cells that become effector T cells and Tregs migrate mostly to secondary lymphoid tissues. On the other hand, Th17 cells transdifferentiate into regulatory T cells during resolution of inflammation.

Th17 cell development is distinct from the development of Th1, Th2, and Treg cells and was characterized by unique transcription factors and cytokine requirements. Th17 cells were reproduced when IL-6, TGF-β, and other inflammatory cytokines are present through T-cell priming [55]. IL-17 induces a number of inflammatory cytokines, neutrophil-attracting chemokines, and inflammatory mediators. For the induction of T-cell proliferation, IL-2 is required and IL-7 and IL-15 drive proliferation of T cells in an antigen-independent manner in lymphopenic conditions [51], [52]. The formation of Th17 cells has been suppressed by IL-2 [53]. For the formation of induced Tregs, FoxP3 and STAT5 are important and for the formation of Tr1 cells, c-Maf and aryl hydrocarbon receptor (AHR) are important [50]. The generation of Tregs and Th17 cells has been modulated by many other factors beyond cytokines. After generation, Tregs and Th17 cells has been migrated and is regulated by trafficking receptors such as chemokine receptors and adhesion molecules [54].

Natural Tregs (FoxP3^+^ T cells) cannot migrate directly into tumors unless tumors are formed in lymphoid tissues as it can migrate to lymph nodes. After gaining the memory or effector-type trafficking receptors, antigen clued-up in secondary lymphoid tissue and FoxP3^+^ T cells can migrate into tumors. All through antigen priming, loss of CCR7 and CD62L occurs which is required for migration of antigen-primed FoxP3^+^ T cells into tumors. Since, induced FoxP3 are downregulated for CCR7 and CD62L as well as upregulated for memory or effector-type trafficking receptors such as CCR4, CCR5, CCR8, CCR10, and/or CXCR4, they can migrate into the tumor via the tumor-draining lymph nodes. Besides, transportation of dendritic cells (DCs) plays important roles in the generation of FoxP3^+^ T cells and Th17 cells in lymph nodes. Furthermore, in tumor-draining lymph nodes, soluble tumor tissue factors are collected and some affect T-cell priming and differentiation happen. Additionally, in tumor microenvironment macrophages (Mac), DCs, and MDSC suboptimally activate T cells which possibly plays significant roles in sustaining the phenotype of FoxP3^+^ T cells and Th17 cells in tumors. In fact, there is no such thing as tumor-specific trafficking receptors, Rather than, T cells inconsistently use conventional trafficking receptors to migrate into different tumors [55].

### 3.8 Tregs and Th17 cells: antitumor immune responses

There is a belief that, if T cells are present in the tumor, there is greatly reliable prognostic factor for survival of cancer patients [56], [57]. We can find much evidence of the strong positive correlation between patient survival and frequencies of memory CD4^+^ T cells and CD8^+^ T cells in many cancer types. Some study shows that oncogenesis had increased in Pan-T-cell- or γδ-T-cell-deficient animals or humans [58]. Amazingly, αβ T cells have a greater positive effect on tumor size but a small negative effect on tumor numbers. This suggests that αβ T cells may even promote tumor growth and are composed of heterogeneous subsets with different functions.

The T cells that suppress antitumor immune responses are FoxP3^+^ T cells and other regulatory T cells. We can say, cells that can inhibit antitumor immune responses and promote tumor growth are FoxP3^+^ T cells [59]. Several FoxP3^+^ T cells are self-reactive and moreover effective in preventing numerous autoimmune diseases. The same function of FoxP3^+^ T cells can be used to stimulate tumor growth. The reason may be, tumor cells primarily express self-antigens, and FoxP3^+^ T cells can efficiently suppress immune responses to self-antigens [60]. Therefore, patient survival rates are inversely correlated with the frequencies of FoxP3^+^ T cells in many tumor types [57], [61]. Nevertheless, some positive correlation or lack of correlation has been reported as well [62].

Some research in a mouse model shows that Th17 cells can promote CD8^+^ T-cell-mediated antitumor immune responses [63]. In addition, antitumor immunity has been increased by the polarization of CD8^+^ T cells into Tc17 cells [64]. Sometimes, to increase antitumor immunity, Th17 cells may become Th1 cells or activate CD8^+^ T cells. Unexpectedly, at early stages of tumorigenesis, Th17 cells can cause inflammation to initiate development of inflammatory tumors.

The occurrences of FoxP3^+^ T cells and Th17 cells replicate the circumstance of the tumor microenvironment, apart from their effector functions. Inflammatory tumors with high expression of inflammatory cytokines would harbor high numbers of Th17 cells whereas, non-inflammatory tumors with low expression of IL-6 and other inflammatory cytokines would have high numbers of FoxP3^+^ T cells. Within the same group of cancers, Tumors are heterogeneous in the tumor microenvironment and it is not wise to say that all tumors fit into the inflammatory *vs.* non-inflammatory tumor model [66]. Both T-cell subsets can be increased or decreased depending on the balance of cytokines and other tissue factors while there is an inverse correlation between FoxP3^+^ T cells and Th17 cells. An example of this situation is invasive ductal breast carcinoma [65].

**Fig. 2:**
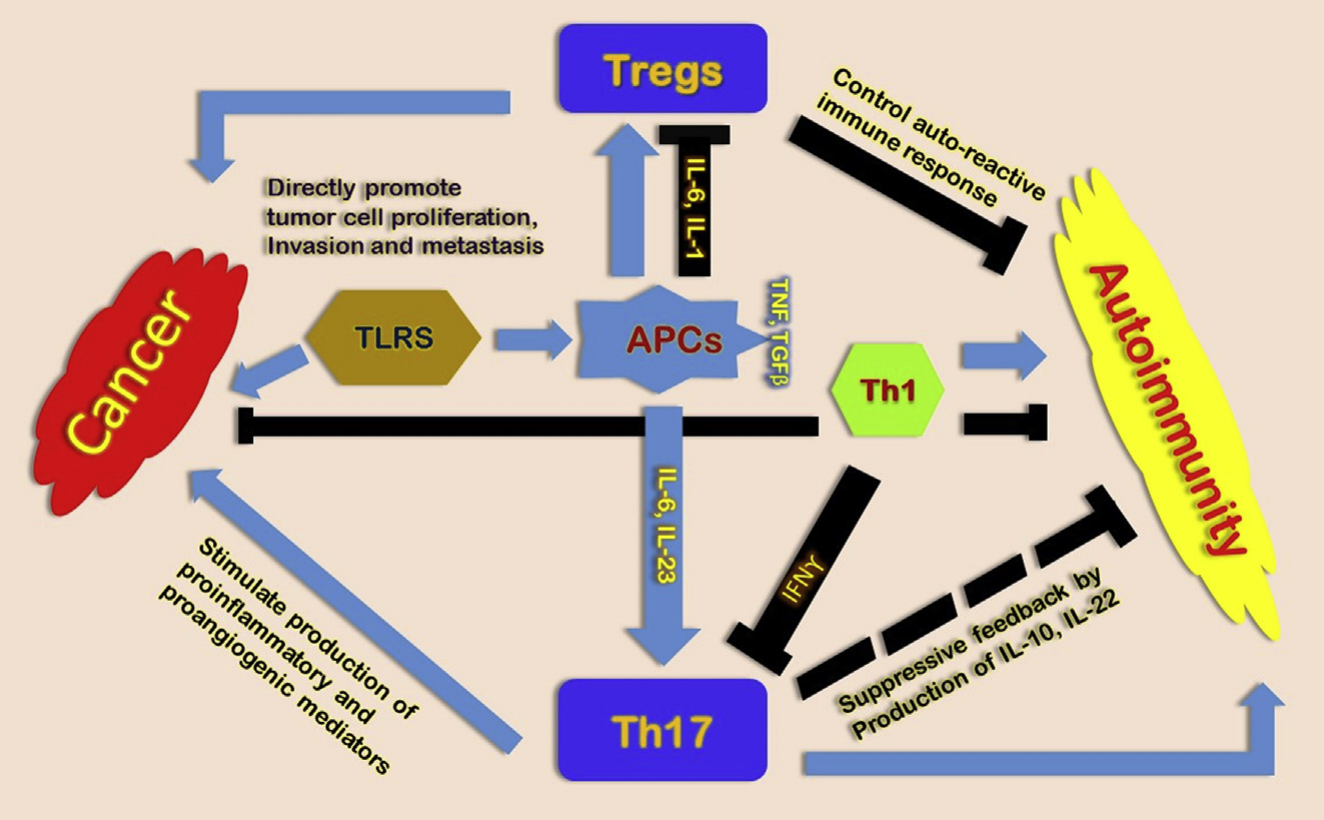
**Role of regulatory T cells, Th17 cells, and TLRs during inflammation, cancer and autoimmunity**. Differentiation of Th1, Th17 and Tregs by producing various cytokines had directed by APCs activation, which earlier activated by TLRs. Therefore, TNF promotes Tregs and TGFβ derived from APC and furthermore IL-1, IL-6 AND IL-23 with TGFβ promote Th17. Induction of Th17 responses induced by TLR agonists and autoimmunity. Moreover, Induction of APC produces IL-12 which promote IFN_ϒ_ producing Th1 cells suppresses Th17 responses and TLR agonists promotes Treg activity. Therefore, Induction of Th17, Th1 and Treg polarization due to activation of TLRs can either favor or counter autoimmunity respectively. Likewise, Activation of TLRs can plays dual role, either can promote tumor development by suppressing immune responses against tumor or trigger anti-tumor immunity [95].

Xin Chen and et al. (2009) describe The Role of Regulatory T Cells, Th17 cells, and TLRs during inflammation, Cancer and autoimmunity (See Fig. 2).

## 4 Tumor and inflammation

One of the seventh hallmarks of tumor development and progression is chronic inflammation which is a consistent feature of the tumor microenvironment [9], [66]. The study shows that among all cancer, 25% of cancers that were associated with chronic inflammation is sustained by infections like hepatitis or inflammatory conditions of diverse origin like prostatitis [9]. Furthermore, even tumors not directly associated with inflammation, cells, and mediators of the inflammatory response are present in the microenvironment [67]. A recent study shows that immune cells can directly contribute to tumor growth, angiogenesis, tumor cell survival, and metastasis [68], [69].

**Fig. 3:**
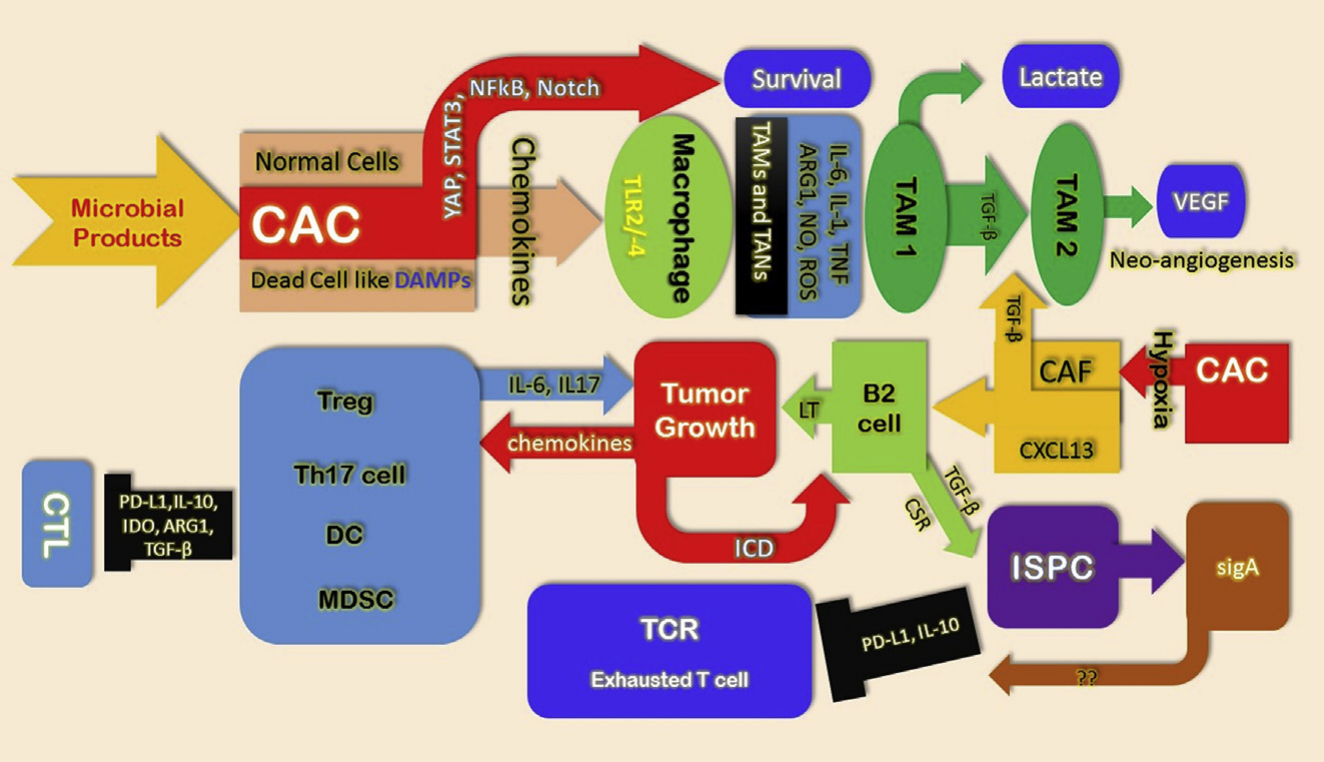
**Relation between inflammation and tumor**. Dead cancer cells (CAC) activate myeloid cells that were recruited into the tumor due to the production of chemokines by CACs, which releases defective barrier associated with early tumors (DAMPs), which allows penetration of microbial products. After that, the cytokines expressed by TAMs and TANs such as IL-1, IL-6, and TNF promote CAC survival and proliferation caused by activation of NF-κB, STAT3, YAP, and Notch. Later, due to insufficient O_2_ supply, the growing tumor secretes lactate and acquires a hypoxic core which causes CAF activation and production of HIF-1-induced TGF-β TAM1 cells to convert to TAM2 phenotype, which produces VEGF to support neo-angiogenesis. Now, CAFs express TGF-β and CXCL13 recruits that convert lymph toxin-producing B2 cells which support further tumor growth. Tumor-promoting Th17 cells, immunosuppressive Tregs, and MDSCs had expressed in the inflamed tumor bed by chemokines. Additionally, induction of exhausted or Anergic-like phenotype in cytotoxic T cells by ISPCs has been class switched recombination (CSR) by tumor-infiltrating B cells [70].

The various processes through which inflammation promotes tumor development described by Shabnam S et al. (2016) briefly (See Fig. 3).

### 4.1 Characteristics of tumor-associated macrophages

The macrophages within a tumor were referred to as tumor-associated macrophages. The TAMs can release a vast diversity of growth factors, proteolytic enzymes, cytokines, and inflammatory mediators upon activation by cancer cells. TAMs promote tumor metastasis through several mechanisms that include tumor angiogenesis, tumor growth, and tumor cell migration and invasion [71]. Poor prognosis of malignant tumors is largely interrelated with the infiltration of macrophages [72]. To facilitate tumor progression, TAMs perform a number of different roles in the tumor microenvironment, and the density of TAMs in human tumors closely associates with poor prognosis [73], [74].

TAMs with majority M2 type macrophages contain some subtypes including M2a, M2b, and M2c [75]. The function of M2 macrophages varied with the subtypes such as subtype M2a inhibit Th1, activate Th2, initiate the immune response inhibiting and killing the parasites; subtypes M2b and M2c have immunosuppressive effects [76]. During certain circumstances, TAMs prompt both M1 and M2 markers relevant to tumor type and the stage of tumor development. For example, increased expression of inducible nitric oxide (iNOS or NOS2, an enzyme expressed by M1 macrophages) together with elevated levels of Arg-1 (usually expressed by M2 macrophages) were observed in TAMs in Meth A – sarcoma, and prostate tumors, CT26 murine colon tumors [77]. The immunosuppressive macrophages are called myeloid-derived suppressor cells (MDSCs). MDSCs are increased in patients with head and neck, breast, non-small-cell lung, and renal cancers. Some of the phenotypes of murine MDSCs are CD11b^+^, Gr-1^+^, IL-4α^+^, F4/80^−^ [78].

### 4.2 Tumor-associated myeloid cells: protumoral functions

Several pieces of evidence have supported that tumor cells secrete factors to – sustain innate immune cells such as, neutrophils, dendritic cells, and monocytes, and develop from a myeloid progenitor cell, i.e. myelopoiesis, accretion, and functional differentiation of myelomonocytic cells. This, in turn, produces T-cell dysfunction and provides essential support for the angiogenesis. The stroma remodeling required for tumor growth [79]. Sica A. et al. (2011) explained mechanisms of TAM recruitment and their protumoral function. Chemokines such as CCL2, CCL5, CCL7, CCL8, CXCL12 and growth factors, for example, M-CSF, PDGF, and VEGF aggressively recruit blood monocytes at the tumor site, where they distinguish in tumor-associated macrophages. Now, Tumor microenvironmental signals, for example, IL-10, PGE2, M-CSF, TGFp, and IL-6 stimulate TAM polarization toward the M2 phenotype. Additionally, TAM promotes tumor growth and progression through various mechanisms. Subsequently, TAM inhibits the antitumor responses by the secretion of immunosuppressive cytokines such as IL-10 and TGFp, and by selective recruitment of naive T cells, via the chemokine CCL18, Th2, and Treg, via the chemokines CCL17 and CCL22. Furthermore, TAM also contributes to both blood and lymphatic neovascular formation by releasing angiogenesis such as VEGF, FGF, TGFp, PDGF, MMPs, and chemokines and lymphangiogenic, for e.g., VEGF-C, VEGF-D factors. Moreover, TAM promotes tumor invasion and metastasis formation through the secretion of matrix proteins like ECM, matrix remodeling enzymes such as MMPs, proteases, cathepsins, uPa, and their activators or chemokines [79].

## 5 Metastasis formation

Most of the malignant solid tumors metastasize from the primary organ to another, for example, the lungs, liver, bone and brain. To establish the metastatic tumor, cancer cells undertake several steps, forming a new tumor at a distant organ site that is known as the metastatic cascade [80].

During early stages of primary tumor development, cancer cells secrete chemokines and cytokines to recruit tumor-associated macrophages (TAMs), tumor-associated neutrophils (TANs), myeloid-derived suppressor cells (MDSCs) and regulatory T (Treg) cells. Then through the production and expression of various factors, including β-galactoside-binding protein (βGBP), programmed cell death 1 ligand 1 (PDL1) and B7-H4, these cells directly suppress the cytotoxic functions of natural killer (NK) cells and CD8^+^ T cells. It helps to increase tumor growth, invasion and way out from the primary site. Accumulation of Treg cells is also stimulated by TAMs, through the production of CC-chemokine ligand 22 (CCL22) and by regulatory B (Breg) cells, through transforming growth factor-β (TGFβ) secretion. Furthermore, MDSCs secrete interleukin-6 (IL-6), IL-23 and TGFβ that recruit T helper 17 (Th17) cells, which secrete IL-17. This promotes recruitment of MDSCs and the secretion of granulocyte colony-stimulating factor (G-CSF) from cancer-associated fibroblasts (CAFs) which in turn promotes the immunosuppressive function of MDSCs. Moreover, the plasmacytoid dendritic cell (pDC)-mediated mechanism has been recruited by MDSCs.

On the other hand, before tumor arrival in the metastatic sites, the primary tumor also produces systemic factors, for example, VEGFA, TGFβ, TNF, and LOX, which induce chemotactic protein expressions like S100A8, S100A9, SAA3 and extracellular matrix remodeling. Now, these deviations recruit immature myeloid cells which form clusters and secrete MMP9 to stimulate subsequent outgrowth of the metastasizing cancer cell. Furthermore, the immature myeloid cells express very late antigen 4 (VLA4) and they also recruited to the pre-metastatic niche by its ligand fibronectin. TAMs and Treg cells are also recruited to the pre-metastatic niche by primary tumor-derived fibrin clots, and by CCL2 and CCL22, respectively, and these cells promote future metastasis [80].

Other mechanisms through which TAM promotes cancer metastasis are the promotion of angiogenesis and lymphangiogenesis, induction of tumor growth, enhancement of tumor cells migration, and invasion. Accumulating evidence has highlighted the importance of the cross-talk between TAM and cancer cells for tumor cells migration [79].

## 6 Recent and future development in TAMs for targeting cancer therapy

A recent study and growing evidence have demonstrated that TAM accumulation in the cancer associated with poor clinical prognosis and resistance to cancer therapy [81], [82]. TAMs are nowadays emerging as attractive targets for therapeutic intervention. Lately, several studies on TAM-targeting cancer therapy have focused on the strategies like (1) inhibition of macrophage recruitment, (2) conversion of the antitumor M1 from pro-tumorigenic M2 phenotype, (3) suppression of TAM survival [7].

Macrophage recruitment into tumors had facilitated by tumor- and stroma-derived chemoattractants. Hence, the inhibition of macrophage recruitment through the modulation of the chemoattractants may possibly become a more effective cancer therapy. Undoubtedly, the inhibition of CCL2 with Bindarit reduced macrophage recruitment which suppressed tumor growth [83]. Subsequently, reduction of macrophage infiltration and tumor growth has been possible by selective inhibition of VEGFR2 with a specific monoclonal antibody [84]. An additional approach for manipulating intratumoral macrophage numbers might be colony-stimulating factor 1 receptor (CSF-1R)-targeted therapy. Inhibition of CSF-1R dimerization has been possible by the humanized monoclonal antibody RG7155. According to Ries (2014), patients with diffuse-type giant cell tumor, the clinical activity of RG7155 was evaluated and was revealed to induce a striking reduction in the CSF-1R + CD163+ macrophage population within tumor tissues [85]. An effective tyrosine kinase inhibitor of CSF-1R such as PLX3397 improved the efficiency of immunotherapy by declining macrophage infiltration and stimulating tumor-infiltrating lymphocytes [86]. Hopefully, macrophage chemoattractants like CCL5 and CXCL12 might be encouraging targets for drug development [87], [88]. Since the classical M1 macrophage possesses antitumor activity, the polarization of tumoricidal M1 from tumor-promoting M2 macrophages seems to be a better possible target for cancer therapy. Numerous studies have reported that tumor-supporting macrophages lead to TLR activation to tumoricidal effectors. Shime and colleagues found in tumor-bearing mice that induction of the assembly of pro-inflammatory cytokines and subsequently acceleration of M1 macrophage polarization has been activated promptly by TLR3/Toll-IL-1 receptor domain-containing adaptor molecule 1 by Poly(I:C) [89]. A clinical drug for cancer therapy such as Zoledronic acid has been found to inhibit spontaneous mammary carcinogenesis by reverting macrophages from the M2 phenotype to the M1 phenotype [90]. Developing evidence has shown that structural and functional abnormalities contain intratumoral blood vessels, which varies the tumor microenvironment and affects the tumor development and responses to cancer therapy. Therefore, enhancement of cancer treatment is possible by the restoration of normal blood vessel structure and function. Furthermore, blockade of the pro-tumorigenic effects of TAMs through vascular normalization could be possible by re-education of TAM within the tumor. Polarization from an M2 to M1 phenotype suppressed mammary tumor growth and angiogenesis *in vivo* had reported by Zhang et al. [91] Lately, Rolny et al. validated that inhibition of the tumor growth by histidine-rich glycoprotein (HRG) and inducing macrophage polarization and vessel normalization via downregulation of the placental growth factor (PlGF) by metastasis [92].

It has generally been believed that improvement of the therapeutic approach to tumors has been possible by suppression of TAM survival. Directly induction of apoptosis in TAMs using chemical or synthetic drugs could be one the effective strategies to kill TAMs. An antitumor drug such as Trabectedin (ET-743) for the treatment of soft tissue sarcoma and relapsed platinum-sensitive ovarian cancer patients can selectively reduce TAMs in tumor patients by activating the extrinsic apoptotic pathway via TRAIL receptors [93]. Meanwhile, Trabectedin not only targets TAM function but also directly affects monocyte/macrophage-mediated host defense [93]. Thus, developing TAM-specific agents would be essential to limit the side effects. Development of a unique peptide M2pep that preferentially binds to M2 macrophages was described by Cieslewicz and colleagues. A recent study in tumor-bearing mice revealed that M2pep carrying a pro-apoptotic peptide selectively killed TAMs and enhanced survival rates in the mice [6], [94].

## 7 Conclusion

Macrophages play an important role in tumors. Depending on the mode of activation, they may promote tumor growth and suppress local immunity, or attack tumor cells and sustain tumor immunity. Immunotherapeutic strategies to combat cancer should incorporate approaches focused on the attraction and polarization of M1 macrophages, as well as on the reprogramming of M2 macrophages to the M1 subset. This requires a well-based understanding of the different subsets of macrophages in human tumors, as well as their interaction with other members of the immune system, including T helper cells.

As discussed earlier, FoxP3^+^ T cells and Th17 cells play both positive and negative roles in regulating antitumor immune responses. Regardless of the presence of these T cells, some tumors still develop and grow. Thus, these T cells by themselves are not sufficient to effectively mount antitumor immune responses.

TAMs have been shown to enhance tumor invasion, migration, and angiogenesis by inflammation. Recent progresses to elucidate the molecular mechanisms of the functions of TAMs opened the new ways to treat cancer patients by reeducating TAMs to be tumor inhibitory cells. Because high TAM infiltration associated with poor prognosis and therapeutic failure in cancer patients, reprogramming of TAM toward an antitumor M1 phenotype, inhibition of TAM recruitment, and suppression of TAM survival may become the foci of promising novel cancer therapies.

## Conflict of interest

The authors report no conflicts of interest.
